# A Collaborative and Sustainable Approach to Multi-Disciplinary Team Building in Critical Care

**DOI:** 10.1186/2197-425X-3-S1-A480

**Published:** 2015-10-01

**Authors:** A Beane, M-L Kong, L Mc Whirter

**Affiliations:** Barts Health NHS Trust, Adult Critical Care Unit, London, United Kingdom

## Introduction

Cohesive teams are the cornerstone of high quality patient care. Our busy 44 bed tertiary referral unit in a major trauma centre created a new program to enhance the culture of team working and management of emergent situations of its complex patients for a newly established workforce in a time of financial austerity.

## Objectives

Establish a forum for interdisciplinary training to build confidence within newly formed clinical teams

• Familiarization with equipment and clinical algorithms

• to close the loop of clinical governance and meet national patient safety recommendations

## Methods

A multidisciplinary, in situ medium fidelity simulation training program was developed and delivered by an interdisciplinary faculty. Clinical scenarios, airway emergencies for this phase of the program, were chosen to reflect both national guidelines [[1–3]] and learning identified through departmental clinical governance investigations.

200 resident clinicians were invited to participate in scenarios, delivered over 12 days on the intensive care unit. Post simulation debriefs identified individual and team development goals; exploring clinical knowledge, practical skills, human factors and team working behaviours. Participant feedback and observations were collated to direct future departmental training.

## Results

### Teamworking

Effective leadership, role expectations and closed loop communication were highlighted as important by candidates. Teams valued the opportunity to learn from mistakes and reported a greater understanding of team working (89%).

### Equipment and algorithm

The need for up-to-date knowledge and practical experience of applying emergency algorithms was highlighted. 72% of respondents reported improved awareness of the difficult airway algorithm. All clinicians valued the exposure to the difficult airway trolley specific to their clinical environment.

### Confidence and relevance

83% of respondents felt more confident in their technical and non-technical skills after the session. 4% found the process of being observed discomforting. 100% felt the sessions were directly relevant to their practice. Participants suggested future scenario topics and over half described it as essential for patient safety.

## Conclusions

This program helped increase staff knowledge and embed key skills essential for the management of airway emergencies, a locally and nationally identified patient safety priority. Teams took the opportunity to foster good communication and team working. Future directions for the program include scenarios involving bleeding patients and the evaluation of knowledge retention by participants after 6 months.
